# Role of apelin as a biomarker in functional recovery and post-stroke-associated sarcopenia: insights from rehabilitation therapy

**DOI:** 10.7717/peerj.20820

**Published:** 2026-03-06

**Authors:** Da-Jung Lee, Dong Kyu Choi, Joong-Gook Kim, Wanil Kim, Hwan-Kwon Do

**Affiliations:** 1 Department of Physical Medicine and Rehabilitation, Haeundae Paik Hospital, Inje University, Busan, Republic of Korea; 2 School of Life Science and Biotechnology, BK21 FOUR KNU Creative BioResearch Group, Kyungpook National University, Daegu, Republic of South Korea; 3 Research Center, Dongnam Institute of Radiological & Medical Sciences (DIRAMS), Busan, Republic of South Korea; 4 Department of Biochemistry, Department of Convergence Medical Science, and Institute of Medical Science, Gyeongsang National University College of Medicine, Jinju, Republic of South Korea; 5 Department of Physical Medicine and Rehabilitation, Dongnam Institute of Radiological & Medical Sciences (DIRAMS), Busan, Republic of South Korea

**Keywords:** Stroke, Sarcopenia, Rehabilitation, Apelin, Muscle mass, Functional recovery

## Abstract

**Background:**

Skeletal muscles play critical roles in mobility, respiratory function, and metabolic regulation by releasing myokines. Age-related sarcopenia, characterized by the loss of muscle mass and function, exacerbates health outcomes, including disability and mortality. Stroke survivors are particularly vulnerable to muscle wasting, known as stroke-related sarcopenia, which affects their recovery and quality of life. This study aimed to investigate the effects of rehabilitation on apelin expression, clinical outcomes, and psychosocial well-being in stroke survivors.

**Methods:**

This single-center observational study enrolled 23 patients with stroke who underwent rehabilitation. Outcome measures included apelin concentration using enzyme-linked immunosorbent assay, cytokine profiling, skeletal muscle index (SMI), phase angle, grip strength, balance, functional scores (Modified Barthel Index, Berg Balance Scale), and psychosocial measures (SF-12, Fatigue Severity Scale). Data were collected at baseline and discharge after 4–6 weeks of rehabilitation.

**Results:**

Apelin levels increased significantly after rehabilitation (46.81–59.23 ng/mL, *p* < 0.001) and correlated with improved SMI (6.67–7.11 kg/m^2^, *p* = 0.0035) and functional outcomes (*p* < 0.001). Significant changes in the cytokine profiles highlighted exercise-induced anti-inflammatory responses. Psychosocial assessments revealed reduced fatigue and improved quality of life (*p* < 0.001).

**Conclusion:**

Stroke patient rehabilitation enhanced apelin expression, skeletal muscle mass, and functional recovery. Therefore, apelin may serve as a biomarker to monitor muscle health and rehabilitation outcomes.

## Introduction

Skeletal muscle plays a pivotal role in physical activity, is associated with respiratory function, and is recognized as the largest endocrine organ that releases myokines that influence metabolism and health (Ogawa & Suenaga, 2021). Sarcopenia is defined as the progressive and generalized age-related loss of skeletal muscle mass, strength, and physical function (Cruz-Jentoft et al., 2019). It is strongly associated with various age-related adverse outcomes, including falls, fractures, physical disability, cognitive frailty, and mortality (Beaudart et al., 2017; Cruz-Jentoft et al., 2019; Manrique-Espinoza et al., 2017; Tolea, Chrisphonte & Galvin, 2018). Most individuals experience a decline in skeletal muscle mass due to aging, which directly and indirectly significantly increases the financial burden associated with disability (Janssen et al., 2004). Consequently, the early detection and appropriate management of sarcopenia, including prevention and treatment strategies, are crucial in rehabilitation settings.

Sarcopenia is not only a concern for the aging population but also significantly affects stroke survivors (Su, Yuki & Otsuki, 2020). Stroke is a globally recognized cause of disability (The GBD 2016 Lifetime Risk of Stroke Collaborators, 2018; Winstein et al., 2016). Individuals who survive a stroke must cope with the long-term effects, including mobility impairments, cognitive and communication difficulties, and emotional challenges (Carod-Artal & Egido, 2009; Scherbakov et al., 2013). Reduced physical activity and functional ability following a stroke often lead to muscle wasting and weakness. Previous studies have referred to these skeletal muscle adaptations as “stroke-related sarcopenia” (Scherbakov, Sandek & Doehner, 2015; Shiraishi et al., 2018; Su, Yuki & Otsuki, 2020). Sarcopenia further exacerbates the physical limitations experienced by stroke survivors, negatively affecting their overall quality of life and restricting their ability to perform daily activities (Shiraishi et al., 2018; Yoshimura et al., 2019).

Despite the underdiagnosis of sarcopenia and the ongoing challenges in its treatment, exercise remains the most important approach for preventing and managing sarcopenia (Dent et al., 2018; Kryger & Andersen, 2007; Yoshimura et al., 2018; Zhao et al., 2022). Rehabilitation offers numerous benefits, including improvements in muscle strength and mass, enhancement of functional performance, reduction of comorbidities, prevention and delay of disease progression, and improvement in quality of life (Lu et al., 2021; Shen et al., 2023). Strategies encompassing both aerobic and resistance exercises are pivotal for optimizing functional recovery and preserving skeletal muscle mass (Iolascon et al., 2014). Rehabilitation programs play a crucial role in helping stroke survivors restore their functional abilities and enhance their overall quality of life. These programs typically aim to restore motor skills and retrain the affected muscles to improve strength and coordination (Winstein et al., 2016). Previous studies have demonstrated that heavy resistance training positively affects muscle volume in very old individuals without a history of stroke (Kryger & Andersen, 2007). However, post-stroke patients often face difficulties with performing heavy-resistance tasks. Additionally, there is a paucity of literature reporting the actual changes in skeletal muscle mass among patients undergoing rehabilitation, and the specific effect of post-stroke rehabilitation on sarcopenia remains poorly understood.

Therefore, this study aimed to examine alterations in apelin expression levels in the blood of individuals who had suffered a stroke and underwent rehabilitation. Apelin was first identified in 1998 as a ligand of the orphan G protein-coupled receptor APJ (Tatemoto et al., 1998). Recently, its role in physiological regulation has garnered attention due to its diverse beneficial metabolic effects, particularly on muscle and stem cell function (Castan-Laurell et al., 2011; Dray et al., 2010; Vinel et al., 2018). With aging, there is a reduction in the synthesis of apelin in the skeletal muscle, which results in decreased plasma apelin levels (Vinel et al., 2018). These findings suggest that apelin may be involved in age-related sarcopenia. Additionally, apelin regulates muscle mass, function, repair, and regeneration (Kwak et al., 2019; Kwon, Moon & Min, 2020). Although the role of apelin has been investigated in general age-related sarcopenia, its specific dynamics in stroke-related sarcopenia and during functional recovery have not been elucidated. This study is among the first to longitudinally evaluate apelin levels in a clinically defined stroke rehabilitation cohort.”

This study primarily aimed to investigate the pre- and post-rehabilitation changes in serum apelin levels in stroke survivors and determine their association with functional recovery measures. Additionally, as an exploratory single-patient sub-study, we profiled inflammatory and growth-related cytokines to provide preliminary insights into the broader biochemical effects of rehabilitation.

## Materials & Methods

### Participants and setting

We conducted a single-center observational cohort study from March 2022 to February 2023 at Inje University College of Medicine, Haeundae Paik Hospital, Busan, Republic of Korea. The study population included all patients who underwent acute stroke treatment and were scheduled for rehabilitation. The exclusion criteria were as follows: previous history of stroke; inability to undergo bioelectrical impedance analysis (BIA), such as patients with cardiac pacemakers whose skeletal muscle mass data from BIA were missing; participation in a rehabilitation program for less than 2 weeks; and comorbid conditions that may affect skeletal muscle mass (*e.g.*, cancer, sepsis, end-stage renal disease, hip fracture, and vertebral compression fracture). We also excluded those who did not provide consent to participate in the study. All participants underwent a rehabilitation program for at least 3 h daily, from the end of acute stroke management until the date of discharge (approximately 4–6 weeks). Rehabilitation was customized based on the physiatrist’s instructions to fit the functional abilities and disabilities of the patients, such as physical therapy, including paralyzed limb facilitation (muscle strength training mainly for leg paralysis), range of motion exercises, basic movement training, walking training, and activities of daily living (ADL) training. All patients were regularly served three meals, provided as oral, intravenous, or enteral nutrition, as determined by the physiatrist. Nutritional management during hospitalization followed standardized hospital dietitian protocols, allowing limited individualization based on each patient’s nutritional and functional status. Daily energy and protein intake targets were set at approximately 25–30 kcal/kg/day and 1.0–1.5 g protein/kg/day according to institutional practice, with adjustments only for patients with clinical indications such as malnutrition or renal impairment. All nutrition plans were supervised by registered dietitians, and no participant received experimental nutrition products or interventions beyond standard care.

### Procedures and assessments

Basic information was obtained at admission from medical records, including age, sex, body mass index (BMI), presence of hemiplegic symptoms, National Institutes of Health Stroke Scale (NIHSS) score, serum albumin level, and time (days) from stroke onset. The NIHSS scores range from 0 to 42, with higher scores indicating more severe neurological deficits. BMI was calculated as weight in kilograms divided by height in meters squared. Laboratory, clinical, and psychosocial outcomes were obtained at T0 (rehabilitation initiation, baseline) and T1 (discharge, post-rehabilitation). The raw data for all clinical parameters are available in the supplementary material provided (see File S1).

### Laboratory assessments

#### Determination of apelin concentration

At baseline and after post-rehabilitation, three mL of blood was collected from each patient. All post-training samples were obtained within 24 h after the final rehabilitation session, most often during the routine laboratory tests conducted on the next morning hours, to minimize the influence of acute exercise effects. The samples were centrifuged to separate plasma and red blood cells, which were stored in individual tubes after treatment with ethylene diamine tetraacetic acid (EDTA). The plasma samples were preserved in a deep freezer at −80 °C until further analysis. The concentration of apelin was determined using an enzyme-linked immunosorbent assay (ELISA) and a human apelin EIA kit (RayBiotech, Peachtree Corners, GA, USA) according to the manufacturer’s instructions. The kit employs anti-apelin antibodies and targets the peptide C-terminus present in all active forms of apelin, including Apelin-36, Apelin-31, Apelin-28, and Apelin-13. Absolute quantification was also performed and recorded through reference verification for each assay.

#### Cytokine array analysis

The collected sera were centrifuged, and the supernatant was analyzed for secreted cytokines and chemokines using a RayBio C-Series Human Cytokine Antibody Array C3 Kit (RayBiotech, Peach Tree Corners, GA, USA) according to the manufacturer’s instructions. All cytokine and chemokine measurements were performed in technical duplicates from the same serum sample to ensure assay reliability. Briefly, the array membrane was incubated with clarified sera, followed by incubation with a biotinylated antibody. The membranes were further incubated with horse radish peroxidase-conjugated streptavidin and visualized. Images were captured using the ChemiDoc MP system (Bio-Rad, Hercules, CA, USA). Quantitative analysis was performed using the ImageJ software with Protein Array Analysis (plugin by Gilles Carpentier). GraphPad Prism software version 10.0.0 (GraphPad Software, Inc., La Jolla, CA, USA) was used for graphical representations. Due to the single-participant nature of this exploratory analysis, inferential statistics were not applied. Instead, data were analyzed descriptively, and relative expression levels were calculated as fold changes comparing post-rehabilitation values to baseline values. The raw data for all assays are available in the supplementary material provided (see File S2).

### Clinical assessments

#### Bioelectrical impedance analysis (BIA)

##### Skeletal muscle index.

Skeletal muscle mass was estimated by multi-frequency BIA with patients lying in a supine position for ≥15 min during the measurement. Subsequently, the skeletal muscle index (SMI) was calculated by dividing the appendicular skeletal muscle mass by the square of height. BIA measurements were conducted in all patients using a segmental multi-frequency bioelectrical impedance analyzer (InBody S10; InBody^®^, Seoul, Korea), according to the manufacturer’s guidelines. The measurements included SMI and the muscle mass of hemiside/non-hemiside upper & lower extremities. SMI serves as a measure of relative muscle mass because absolute muscle mass is correlated with height (Kim et al., 2018). BIA is a reliable tool for assessing body composition and correlates well with dual-energy X-ray absorptiometry (Kim et al., 2015).

##### Phase angle.

The phase angle (PA) is defined as the ratio of resistance (intracellular and extracellular resistance) to reactance (cell membrane-specific resistance) and is expressed as an angle (Norman et al., 2012). It serves as an indicator of cell membrane function and is commonly used for nutritional assessments and to investigate the risk of various diseases, such as locomotive syndrome. Additionally, the phase angle is considered a useful parameter for assessing frailty in older patients, with a low phase angle being a significant risk factor for frailty (Tanaka et al., 2019). It was measured by using the whole-body phase angle at a frequency of 50 kHz frequency, which is the most commonly used method.

#### Functional assessments

##### Hand grip strength.

Grip strength, a diagnostic criterion for sarcopenia, was evaluated using a handheld dynamometer (Jamar^®^ smart hand dynamometer; Patterson Medical Ltd., Sutton-In-Ashfield, UK). Participants were instructed to sit with their elbow flexed at 90° and their forearm in a neutral position (Roberts et al., 2011), gripping the dynamometer as forcefully as possible for 3 s. Three trials were conducted on both the hemiside and non-hemisides, and the maximum grip strength values were recorded.

##### Berg balance scale.

The Berg balance scale (BBS) is a test with high validity and reliability for measuring functional balance (Miranda-Cantellops & Tiu, 2024). It evaluates both dynamic and static balance by using 14 mobility tasks. Each task was graded on a 5-point ordinal scale ranging from 0 to 4, resulting in a maximum total score of 56. Higher scores indicated better functional balance.

##### Modified Barthel index.

Among the various methods for assessing ADLs, the modified Barthel index (MBI) is a well-established, patient-centered outcome measure commonly used in rehabilitation settings (Pournajaf et al., 2023). It is used to evaluate the functional status of patients both at admission and discharge. The MBI comprises 10 items investigating 10 functional ADLs: feeding, personal hygiene, bathing, dressing, chair-bed transfer, toileting, bladder continence, bowel continence, ambulation or wheelchair use, and stair climbing. Each item has its scale, which is added to yield a total score ranging from 0 to 100, with lower scores indicating more severe functional dependency. Each item was scored by trained physiotherapists who observed the patients while performing functional tasks and evaluated the amount of assistance the patient required using a 5-point scale.

##### Motricity index.

The motricity index (MI), a short test for the assessment of motor function after stroke, was evaluated using three arm movements: pinch grip, elbow flexion, and shoulder abduction, and three leg movements: ankle dorsiflexion, knee extension, and hip flexion (Demeurisse, Demol & Robaye, 1980). The total “arm score” was the sum of three arm test scores; the total “leg score” was the sum of three leg test scores; and the total “side score” was the sum of the arm and leg scores, divided by two. An extra point was added to each limb score such that the highest score was 100% (Collin & Wade, 1990). The authors confirm that the instrument used in this study are available for academic research without requiring explicit permission from the copyright holders.

### Psychosocial assessments

#### 12-item Short Form Survey

The 12-item Short Form Survey (SF-12) is one of the most widely used instruments for assessing self-reported health-related quality of life (HR-QoL) (Ware Jr, Kosinski & Keller, 1996). Originally developed from the 36-item Short-Form Health Survey (SF-36) (Naess et al., 2006), it covers the same eight health domains as the SF-36, with substantially fewer questions, making it suitable for populations with limited attention spans or cognitive challenges (Huo et al., 2018; Naess et al., 2006), such as patients with stroke. The SF-12 comprises a subset of 12 items and and yields two composite measure: the Physical Component Summary (PCS) and Mental Component Summary (MCS) scores, each ranging from 0 to 100, with higher scores indicating better perceived health status. The authors have permission to use this instrument from the copyright holders.

#### Fatigue Severity Scale

The 9-item Fatigue Severity Scale (FSS) is a commonly used self-report questionnaire for measuring the severity of fatigue and its effect on patients’ activities and lifestyle (Krupp et al., 1989). Each score ranged from 1 (strongly disagree) to 7 (strongly agree) and was then added to yield a total score ranging from 9 to 63. The authors confirm that the instrument used in this study are available for academic research without requiring explicit permission from the copyright holders.

### Statistical analysis

GraphPad Prism software version 10.0.0 (GraphPad Software, Inc., La Jolla, CA, USA) was utilized for statistical analyses of all laboratory assessments and to prepare graphical representations. Statistical significance was determined using paired *t*-tests, and results are presented as means and SEM. Significance was defined as a *p*-value less than 0.05.

All statistical analyses in clinical and psychosocial assessments were performed using SPSS software (version 25; IBM Inc., Armonk, NY, USA). Continuous variables are described as mean ± standard deviation, while categorical variables are expressed as the absolute number of patients and percentages. Normally distributed variables were compared using the *t*-test, and skewed variables were assessed using the Wilcoxon signed-rank test. Multiple linear regression analysis was to identify baseline parameters that affect apelin expression. Regression results are as beta coefficients with 95% confidence intervals, and model diganostics-including residual analysis and multicollinearity checks-were performed to confirm model validity.

Given the modest sample size (*n* = 23) and multiple comparisons, Type I error risk was minimized by defining a single primary endpoint (change in apelin) and limiting the number of secondary analyses. Covariate selection for regression models was restricted based on the theoretical relevance and events-per-variable considerations to avoid overfitting.

### Ethical considerations

The study was conducted in accordance with the guidelines of the Declaration of Helsinki and approved by the Institutional Review Board of Inje University Hospital (approval no. 2023-09-011). All the participants provided written informed consent after receiving a detailed explanation of the study protocol.

## Results

### Patient demographics

**Figure 1:**
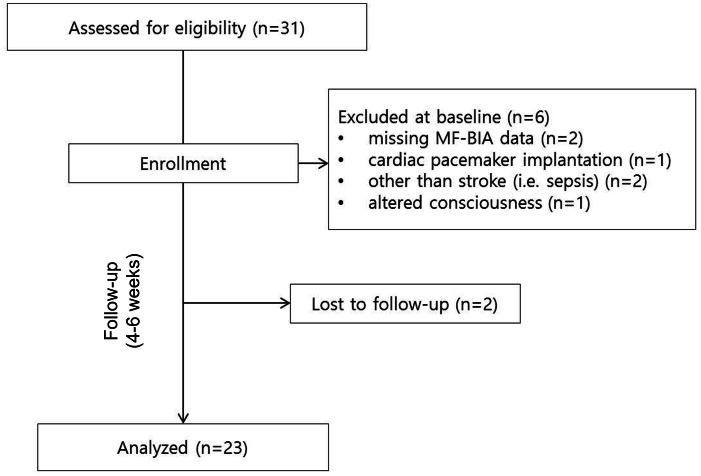
Flowchart of the study population. A total of 31 patients were initially enrolled based on their eligibility for the study. Six patients were excluded due to their condition and lack of assessment. Two patients were lost to follow-up. Finally, 23 stroke survivors were enrolled and completed the full 4–6 week rehabilitation protocol and assessment. Abbreviation: MF-BIA, multi-frequency bioelectrical impedance analysis.

During the study period, a total of 31 patients who met the study inclusion criteria were enrolled. Among these, patients with incomplete initial BIA data (*n* = 2), pacemaker insertion (*n* = 1), altered consciousness during rehabilitation (*n* = 1), or other comorbidities associated with secondary sarcopenia (*e.g.*, sepsis and cancer) (*n* = 2) were excluded from the analysis. Two patients were also excluded due to isolation following coronavirus disease 2019 infection after receiving less than 2 weeks of rehabilitation. Consequently, 23 patients were included in the final analysis (Fig. 1).

The baseline characteristics of the enrolled patients are shown in Table 1. The mean age was 67.13 ± 14.61 years. Of the patients, 56.5% (*n* = 13) were male and 43.5% (*n* = 10) were female. Among all the patients, 87% (*n* = 20) were diagnosed with ischemic stroke, and 13% (*n* = 3) had hemorrhagic stroke. The mean NIHSS score was 8.69 ± 6.54, and the average hospital stay was 35.61 ±8.35 days. Mean daily protein intake was 1.06 ± 0.12 g/kg/day, and no significant differences between patients were observed in nutritional targets or energy provision during hospitalization.

**Table 1:** Baseline characteristics of study participants.

**Characteristics (*n* = 23)**	
Age (years)	67.13 ± 14.61
Sex, n (%) (Male/Female)	13 (56.5%)/10 (43.5%)
Height (cm)	166.08 ± 8.11
Body weight (kg)	67.21 ± 11.87
BMI (kg/m^2^ )	24.27 ± 3.46
NIHSS	8.69 ± 6.54
Hemiplegic symptom, n (%)	
With hemiplegia/Without hemiplegia	20 (87%)/3 (13%)
Stroke type	
Ischemic/hemorrhagic	20 (87%)/3 (13%)
Days from onset	13 ± 15.56
Length of hospital stay	35.61 ± 8.35
Albumin (g/dL)	3.89 ± 0.43
Protein intake (g/kg/day)	1.06 ± 0.12

**Notes.**

Values are presented as mean ± SD or as number (%).

BMI body mass index NIHSS National Institutes of Health Stroke Scale

### Apelin expression and clinical outcomes

A previous study has shown that the amount of apelin in the blood is closely related to muscle volume and function during the development of age-related sarcopenia (Yoshimura et al., 2019). In this study, we obtained blood samples from patients with stroke before and after rehabilitation to measure the amount of apelin in their plasma. We then correlated these results with improvements in physical function and psychological changes in the patients after rehabilitation.

**Figure 2:**
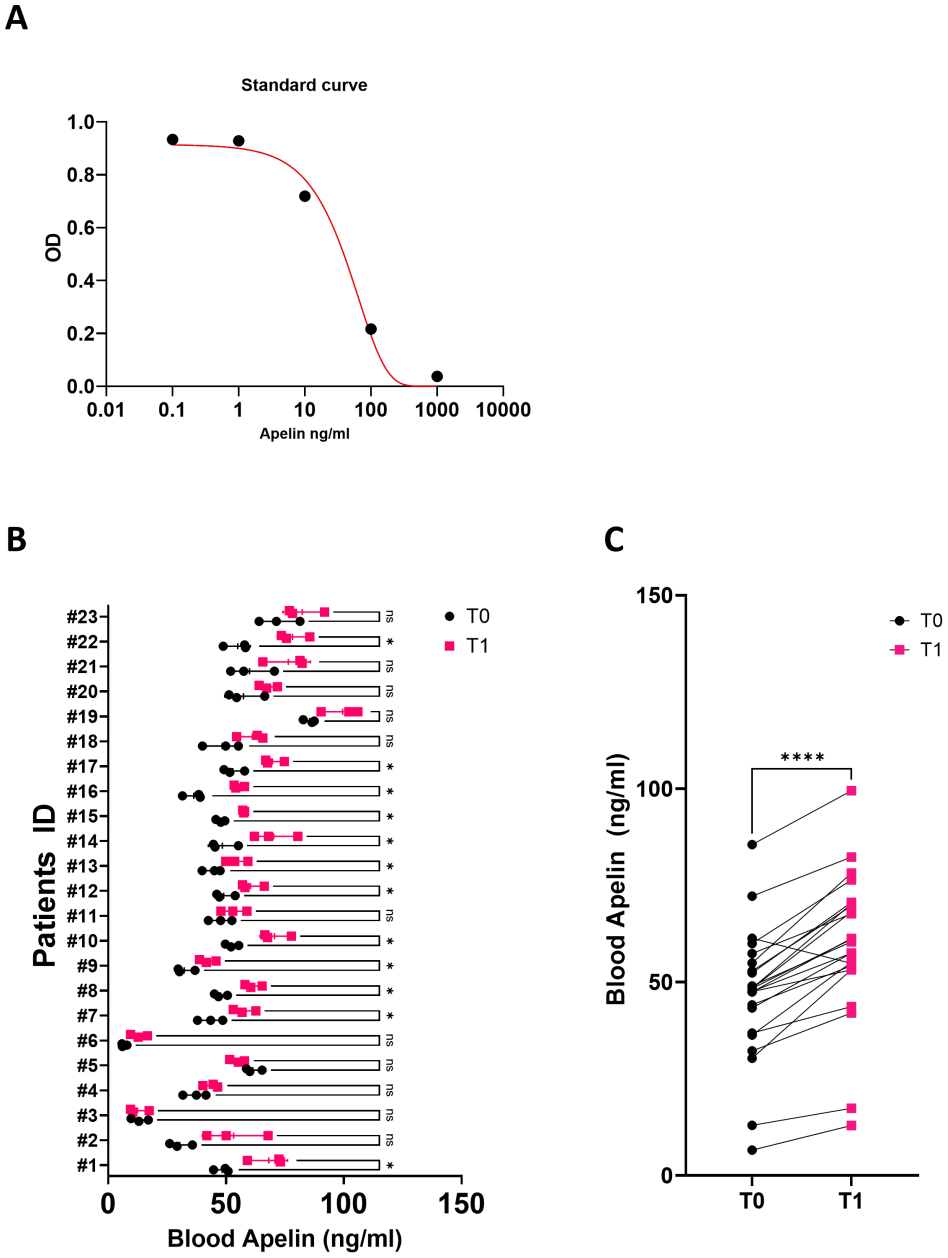
Protein amount of blood apelin after rehabilitation. (A) A standard curve was generated with known amounts of apelin proteins using ELISA. (B) A total of 23 stroke patients with a diagnosis of stroke were enrolled in the study, and blood apelin levels were analyzed at two time points: at pre-rehabilitation (T0) and after completion of the 4–6 week rehabilitation program (T1). All post-training samples were collected within 24 h of the final rehabilitation session, usually during routine laboratory testing the following morning, to minimize acute exercise effects. ELISA was used to determine the specific concentrations of the proteins present in the serum of each individual patient. The statistical analysis revealed that 12 patients exhibited a significant increase in apelin levels after the completion of the rehabilitation program. (C) The blood apelin levels of all patients before and after the rehabilitation program were analyzed. Statistical significance was determined with ns (non-significant), ^∗^*p* < 0.05, ^∗∗∗∗^*p* < 0.0001. Abbreviation: ELISA, enzyme-linked immunosorbent assay.

To achieve this, we first used an ELISA kit that measured the absolute amount of the target protein through a specific antigen-antibody reaction, and a standard curve was constructed using a fixed amount of apelin protein (Fig. 2A). The standard curve obtained by varying the concentration of a set amount of apelin protein showed a concentration-dependent difference in optical density. The level of unknown blood apelin was then determined by measuring the optical density of the patient’s blood (Fig. 2B). Baseline (T0) blood samples were collected at the start of rehabilitation, and post-training (T1) samples were obtained within 24 h after the final rehabilitation session. Blood samples were stored at −80° C immediately after collection and analyzed concurrently with the samples collected after the 1-month rehabilitation exercise program.

The apelin expression level significantly increased from baseline to discharge (from 46.8–59.4 ng/mL, *p* < 0.0001, paired *t*-test) (Fig. 2C). The SMI also significantly increased at discharge compared with baseline (from 6.67–7.11 kg/m^2^, *p* = 0.0035). Additionally, there were significant improvements in hemiside hand grip strength, MBI, BBS, and MI (all *p* < 0.001) (Table 2). Although the Phase Angle (PA) value increased, the increase was not statistically significant. These results suggest that an increase in apelin correlates with enhancements in muscle function and activities of daily living.

Of the 23 enrolled patients with stroke, a statistically significant increase in blood apelin protein levels following the rehabilitation program was observed in 12 patients (Fig. 2C). The MBI increased in all 12 patients, indicating improved physical function. Excluding the five patients who had zero motor power both at baseline and discharge, the remaining seven patients demonstrated an increase in hemi-side hand grip strength. Additionally, after the rehabilitation program, 91% (11 patients) of the patients had increased SMI and BBS scores, reflecting improvements in skeletal muscle mass and physical function. Therefore, changes in the amount of apelin protein in the blood of patients with stroke can serve as an indicator of improved physical function and skeletal muscle mass.

In this analysis, the fold-change in apelin was significantly associated with improved MBI scores. Among the 12 patients who showed a statistically significant increase in apelin levels, the apelin fold-change was 1.34, and the MBI fold-change was 1.41. In three patients with the greatest increase in apelin fold change (1.43), the MBI fold change was 1.6. In the overall cohort of 23 patients, the apelin fold change was 1.27, and the MBI fold change was 1.33, suggesting a significant association between apelin expression and physical function improvement in this patient group. Additionally, the BBS fold-change for the 12 patients with a significant increase in apelin level was 2.4, which was notably higher than the BBS fold-change of 1.98 observed in the total group of 23 patients, indicating a marked improvement in balance abilities. This further emphasizes that apelin expression reflects improvements in physical function.

**Table 2:** Paired within-subject changes in outcome measures between admission (T0) and discharge (T1).

	**At admission (T0)**	**At discharge (T1)**	**Diff_M±SD**	***P*** ** value**
Apelin (ng/mL)	46.81 ± 16.86	59.23 ± 19.47	12.42 ± 7.27	<0.0001
SMI (kg/m^2^ )	6.67 ± 1.03	7.11 ± 1.28	0.44 ± 0.65	0.0035
Phase angle (’)	4.49 ± 1.09	4.91 ± 1.38	0.42 ± 1.26	0.125
Hemiside hand grip (kg)	6.35 ± 9.05	9.05 ± 10.08	2.70 ± 3.04	0.0003
Non-hemiside hand grip (kg)	21.30 ± 12.55	23.73 ± 12.67	2.43 ± 3.28	0.002
MBI	44.26 ± 21.27	58.95 ± 23.55	14.69 ± 10.24	<0.0001
BBS	20.00 ± 14.82	30.22 ± 45.28	10.22 ± 9.17	<0.0001
FSS	42.04 ± 16.07	31.00 ± 15.13	−11.04 ± 13.08	0.001
SF-12 PCS	28.55 ± 7.04	30.76 ± 7.43	2.21 ± 2.70	0.001
SF-12 MCS	33.68 ± 8.57	36.31 ± 8.51	2.63 ± 3.67	0.002
Motricity Index	52.70 ± 19.48	62.33 ± 16.46	9.63 ± 7.13	<0.001

**Notes.**

Values are presented as mean ± SD or as number (%).

SMI Skeletal Muscle mass Index SMM Skeletal Muscle Mass UEx Upper Extremity LEx Lower Extremity MBI Modified Barthel Index BBS Berg Balance Scale FSS Fatigue Severity Scale SF-12 PCS Short Form-12 Physical Component Summary SF-12 MCS Short Form-12 Mental Component Summary

### Cytokine analysis

**Figure 3:**
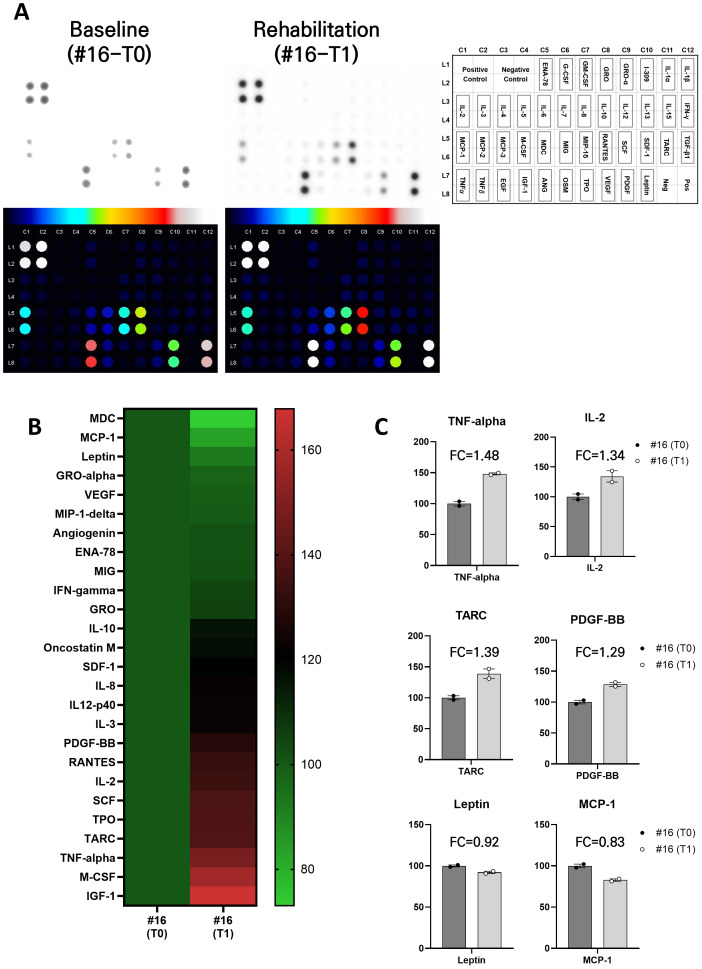
Exploratory blood cytokine analysis of a patient with the largest apelin change after rehabilitation. One patient (patient ID #16) showed a 1.41-fold change in blood apelin after rehabilitation exercises, and their blood was analyzed for various cytokines. (A) Cytokine array membrane images and corresponding reference map. The left and middle panels show the original membrane images from the cytokine array representing pre- and post-rehabilitation conditions, respectively. Each cytokine spot is heat-mapped and labeled at its precise grid position (RayBio C-Series layout: rows 1–6, columns 1–12). The right panel displays a reference grid with cytokine labels only and no background signal, allowing easy cross-identification of each spot. (B) Heatmap showing relative expression of 26 cytokines that displayed adequate expression in the chemiluminescent detection before and after the rehabilitation program. (C) Relative expression levels of 6 selected cytokines that exhibited distinct changes relative to baseline. Bars represent changes in significant cytokines after 1 month of rehabilitation. For each bar, the paired dots indicate the range of technical replicate values derived from the same serum sample. Due to the single-participant design (*N* = 1), inferential statistics were not applied. FC shows the fold changes. Abbreviations: TNF-*α*, Tumor necrosis factor-alpha; IL-2, Interleukin-2; TARC, Thymus and activation-regulated chemokine; PDGF-BB, Platelet-derived growth factor-BB; MCP-1, Monocyte chemoattractant protein-1.

To explore the immunological context of the sharp apelin increase, we performed an exploratory cytokine/chemokine array on the serum collected from the single patient who exhibited the greatest apelin rise (patient #16; Fig. 3A). Exercise is known to provoke a biphasic cytokine response—an acute, transient surge of pro-inflammatory mediators followed by longer-term anti-inflammatory adaptation—whose magnitude depends on exercise type, intensity, and individual characteristics (Docherty et al., 2022; Małkowska & Sawczuk, 2023).

In this patient, exploratory profiling suggested that more than 20 cytokines and chemokines changed directionally after the 4-week rehabilitation program (Fig. 3B). Among them, six (TNF-*α*, IL-2, TARC, PDGF-BB, leptin, and MCP-1) exhibited distinct changes in iexpression levels relative to baseline (Fig. 3C), potentially illustrating a transient activation phase that likely precedes or parallels systemic anti-inflammatory remodeling. These preliminary data exemplify the dynamic immune milieu accompanying rehabilitation-induced functional recovery in this patient with post-stroke-sarcopenia.

### Clinical and psychosocial outcomes

**Figure 4:**
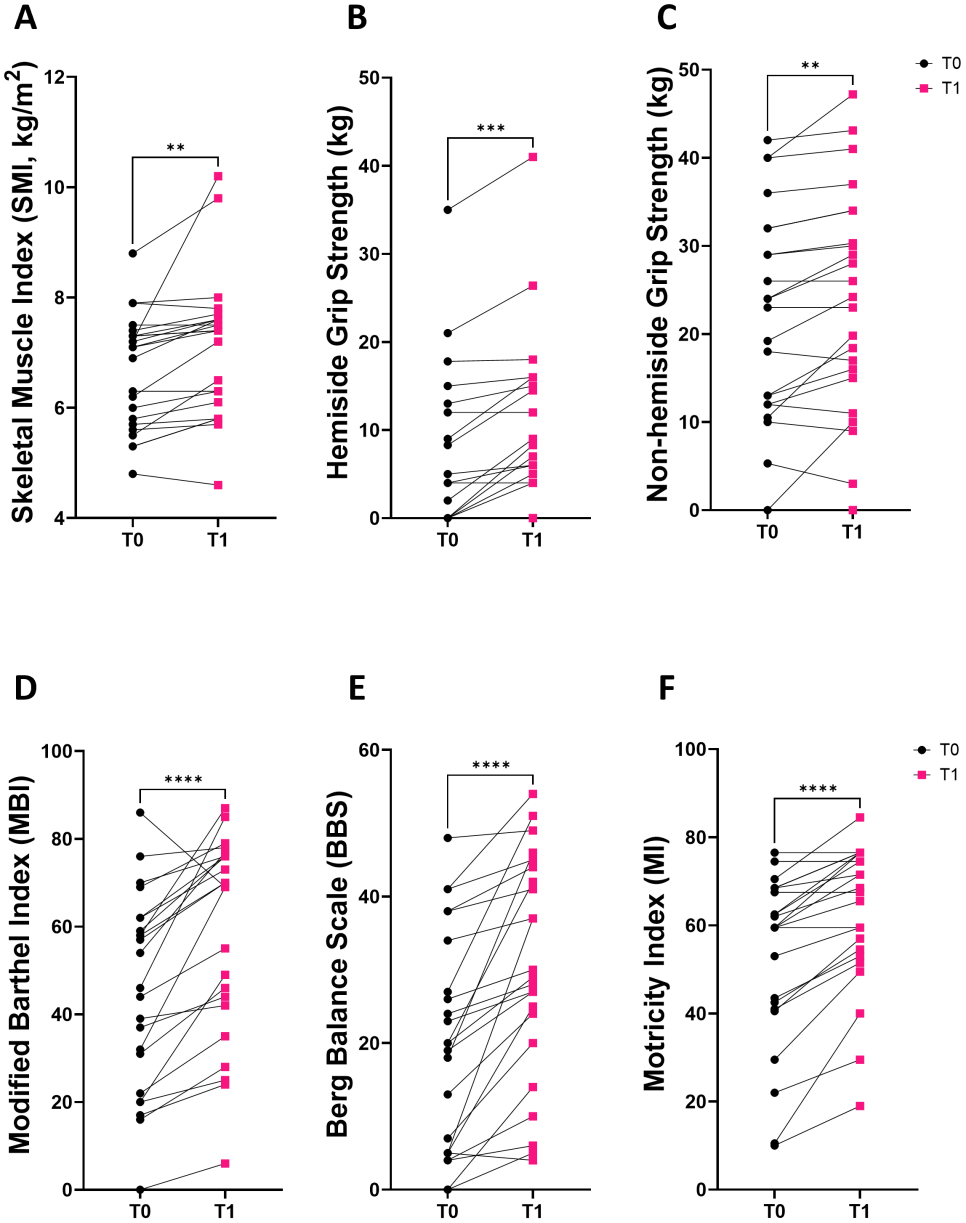
Change of clinical parameters after rehabilitation. These clinical parameters show statistically significant changes after rehabilitation in stroke survivors. (A) Skeletal muscle index(SMI, kg/m 2), (B) hemiside grip strength (kg), (C) non-hemiside grip strength (kg), (D) modified Barthel index(MBI), (E) Berg balance scale(BBS), and (F) motricity index (MI). Statistical significance was determined using paired *t*-tests or Wilcoxon signed-rank tests with ^∗^*p* < 0.01, ^∗∗^*p* < 0.005, ^∗∗∗^*p* < 0.0005, ^∗∗∗∗^*p* < 0.0001.

A comparison of the clinical and psychosocial outcomes between baseline (at the start of rehabilitation) and discharge is presented in Table 2. This comparison is also illustrated in graphical form in Figs. 4A–4F. We observed a statistically significant increase in both the SF-12 physical and mental component scores (PCS:28.5→30.7, *p* = 0.001; MCS: 33.6→36.3, *p* =0.002) following rehabilitation. Additionally, there was a significant decrease in the FSS (42.0–31.0, *p* = 0.001) (Table 2).

These improvements in the SF-12 scales indicate that rehabilitation positively affects both physical function and mental well-being in stroke survivors. The overall decrease in FSS reflects a reduction in fatigue levels, which may facilitate greater engagement in daily activities and enhance the patients’ quality of life.

The PCS correlated significantly with physical function measures such as MBI, BBS, and MI, whereas the MCS correlated with SMI and PA, suggesting a potential link between muscle recovery and psychological improvement (Table 3).

### Association between apelin concentrations and clinical and psychosocial outcomes

Multiple linear regression analysis was performed to evaluate the influence of each baseline parameter on increased apelin expression (Table 4). The model was appropriate (*F* = 9.293, *p* = 0.001; adjusted *R*^2^ = 0.430). Regression coefficients (*β*) with 95% confidence intervals are reported.Higher baseline albumin levels were positively associated with apelin increase (*β* = 15.628, *p* < 0.001), whereas lower baseline MBI was inversely associated (*β* = −0.252, *p* = 0.005). Therefore, albumin exerts a relatively greater influence on apelin concentration, as indicated by its higher beta value than MBI and other parameters. Model diagnostics, including residual and multicollinearity checks, confirmed absence of overfitting. However, no significant associations were observed between increased apelin expression and baseline SMI, PA, grip strength, BBS, or psychological parameters.

## Discussion

In this study, we examined the changes in apelin expression levels and functional, clinical, and psychosocial outcomes in patients undergoing rehabilitation after stroke. The major study findings were: first, apelin levels, skeletal muscle mass, hand grip strength, MBI, BBS, FSS, SF-12 component scores (PCS and MCS), and MI all significantly increased following rehabilitation. Second, greater changes in apelin were associated with more pronounced improvements in physical function indicators such as MBI and BBS. To our knowledge, this is the first human study to demonstrate a significant correlation between changes in apelin levels and improvements in both muscle mass and functional recovery in stroke-related sarcopenia, supported by multimodal assessment including psychosocial scales and cytokine profiling.

**Table 3:** Correlation analysis on clinical and psychosocial outcomes.

	MBI	BBS	MI	SMI	PA
SF-12 PCS	−0.439^*^	0.595^**^	0.515^*^	−0.335	−0.061
SF-12 MCS	0.168	−0.027	−0.045	0.432^*^	−0.527^**^

**Notes.**

Correlation coefficients are presented for relationships between physical and psychosocial parameters.

MBI Modified Barthel Index BBS Berg Balance Scale MI Motricity Index SMI Skeletal Muscle mass Index PA Phase Angle SF-12 PCS Short Form-12 Physical Component Summary SF-12 MCS Short Form-12 Mental Component Summary

* *P* = 0.05.

** *P* = 0.01.

**Table 4:** Multiple linear regression analysis on factors associated with serum apelin change.

	Unstandardized Coefficients		Standardized coefficients	*t*	Sig.	VIF
	B	Std. Error	*β*			
(Constant)	−36.409	11.995		−3.035	0.007	
Albumin	15.628	3.637	0.939	4.297	0.000	1.841
MBI	−0.252	0.080	−0.690	−3.159	0.005	1.841
F(*p*)	9.293(0.001)					
Adj. R^**2**^	0.430					
Durbin-Watson	1.880					

**Notes.**

Regression results are reported as unstandardized (B) and standardized (*β*) coefficients, with 95% confidence intervals (CIs).

Model diagnostics confirmed that regression assumptions were met, including normality and homoscedasticity of residuals, linearity, and absence of multicollinearity (all VIF <  5).

B unstandardized regression coefficient *β* standardized regression coefficient VIF Variance Inflation Factor MBI Modified Barthel Index Adj. R^2^ the adjusted coefficient of determination

Despite the growing interest in stroke-related sarcopenia, the Asian Working Group for Sarcopenia reported that up to 50% of post-stroke patients are diagnosed with this condition (Chen et al., 2020), and there is a lack of information regarding changes in skeletal muscle mass and clinical outcomes after rehabilitation in patients (Yoshimura et al., 2019). Previous studies have highlighted the beneficial effects of heavy resistance training in improving muscle mass in older individuals without a history of stroke. Kryger & Andersen (2007) reported significant increases in muscle volume in individuals aged 85–98 years after a 12-week dynamic resistance training program for knee extensors and flexors. However, high-intensity resistance training poses a challenge for many stroke survivors. Ryan et al. (2011) demonstrated that thigh muscle volume increased in both the paretic and nonparetic limbs of 15 stroke survivors aged 50–76 years after 12 weeks of high-volume, high-intensity leg resistance training. In a recent study by Takahiro et al., patients with stroke aged >80 years participated in rehabilitation programs similar to ours, which included resistance training and ADL training tailored to each patient’s ability (Ogawa & Suenaga, 2021). Significant increases in the skeletal muscle mass and functional improvements were observed in these patients. Similarly, our study demonstrated significant improvements in skeletal muscle mass among patients with stroke, particularly those in the 60–80 age range. Only three patients (13%) did not have an increase in SMI. These results suggest that even patients who struggle with heavy resistance training after stroke can achieve positive outcomes in terms of skeletal muscle mass and functional independence through tailored rehabilitation including balance training, bodyweight exercises, and gait training.

This study is noteworthy for its association with improvements in skeletal muscle mass, functional changes in patients with stroke undergoing rehabilitation therapy, and increased apelin expression. While previous animal studies have demonstrated that exercise-induced myokine apelin promotes muscle regeneration and positive regulatory feedback (Kwak et al., 2019; Vinel et al., 2018), few studies have analyzed the changes in apelin before and after rehabilitation therapy in humans. Vinel et al. (2018) found that supplementation of aged mice with daily injections of apelin *via* adenovirus-mediated gene transfer in a skeletal muscle model improved muscle capacity and myofiber hypertrophy. Additionally, Besse-Patin et al. (2014) reported that when 11 obese men underwent an 8-week endurance training program, muscle apelin mRNA levels increased two-fold. In our study, significant improvements were observed in apelin expression levels after rehabilitation as well as in the SMI and functional levels of patients. Notably, changes in apelin levels were significantly associated with improved physical function.

We performed an exploratory cytokine and chemokine analysis on the single participant who exhibited the greatest increase in apelin levels. In this individual, the increase in apelin was accompanied by observable changes in inflammatory markers. The post-training blood sample in this study was collected from the patient the next morning following the final rehabilitation session, typically within 24 h after completion of the program. This timing was intended to minimize the influence of acute exercise-induced cytokine fluctuations. Therefore, the observed changes are more likely to reflect chronic adaptations to the 4–6 week rehabilitation program rather than transient post-exercise responses in this individual. In this analysis, we found a significant increase in tumor necrosis factor (TNF)-alpha, among others (Fig. 3C). TNF-alpha is a pro-inflammatory cytokine that regulates immune cell activation and prostaglandin synthesis in response to exercise (Docherty et al., 2022). Consistent with previous reports (Kaya, 2016; Rhind et al., 1996), we observed elevated interleukin-2 secretion in this patient in response to rehabilitation. Thymus and activation-regulated chemokine (TARC), also known as CCL17, is a chemokine, a type of signaling protein involved in immune cell migration and activation (Lupancu et al., 2023). TARC is produced by various cell types, including dendritic cells, macrophages, and certain epithelial cells, in response to inflammatory signals. It attracts specific subsets of immune cells, such as T cells expressing the chemokine receptor CCR4, to sites of inflammation or infection. There have been no reports of changes in TARC secretion caused by rehabilitation exercises. However, in this study, we found that TARC protein expression was higher after a 1-month rehabilitation exercise program in the patient with stroke. Platelet-derived growth factor-BB increases during aerobic exercise but decreases during low-intensity exercise (Hashida et al., 2023; Wang, Yi & Tang, 2021). In this patient, its secretion was higher post-rehabilitation, consistent with the results of previous studies. Leptin is a hormone primarily produced by adipose (fat) tissue and plays a crucial role in regulating energy balance and appetite. The relationship between physical exercise and leptin secretion is complex and can be influenced by various factors, including intensity, duration, and frequency of exercise, as well as individual differences such as body composition and fitness level (Bouassida et al., 2006; Kraemer, Chu & Castracane, 2002). Regular physical activity may lead to long-term alterations in leptin levels (Fedewa et al., 2018). In our observation, leptin levels showed a reduction after rehabilitation. MCP1, also known as monocyte chemoattractant protein-1, is a cytokine whose expression decreases in response to exercise (Trøseid et al., 2004). Additionally, we observed lower levels of its secretion in the rehabilitated patient.

Another finding of this study was the significant reduction in fatigue and improvement in HR-QoL observed after rehabilitation. Post-stroke fatigue and depression are common psychosocial challenges faced by stroke survivors and are major factors hindering functional recovery and quality of life (Espárrago Llorca et al., 2015; MacIntosh et al., 2017; Paciaroni & Acciarresi, 2019). Although previous studies investigated pharmacological and non-pharmacological interventions for post-stroke fatigue and depression, a recent Cochrane review concluded that there is insufficient evidence to support specific treatments (Paciaroni & Acciarresi, 2019). The significant improvement in HR-QoL and the reduction in fatigue after rehabilitation therapy observed in this study suggest the potential psychological benefits of such interventions. Additionally, rehabilitation therapy may enhance motivation and functional improvement.

Additionally, we reported that baseline albumin levels and MBI influenced changes in apelin concentrations. Specifically, higher baseline albumin levels, which reflect the nutritional status of the patients, were associated with increased apelin levels. This finding indicates that ensuring a solid nutritional foundation for patients with stroke could positively affect apelin levels after rehabilitation, potentially leading to significant improvements in both sarcopenia and functional outcomes. Notably, a reduction in the initial MBI, which reflects functional status, was also linked to increased apelin levels, further supporting the importance of nutritional and functional factors in recovery.

This study had several limitations. First, it was conducted at a single institution and the sample size was small, which may limit the generalizability of the results. Further studies with larger sample sizes will help elucidate the effects of the intervention in broader settings. Moreover, given the limited sample size and multiple statistical comparisons performed, the possibility of Type 1 error (false-positive findings) cannot be excluded. Therefore, the statistical significance of the observed associations should be interpreted with caution. In addition, cytokine profiling was performed in only one participant, precluding any meaningful group-level inference regarding the relationship between apelin and other inflammatory mediators. Future studies should include systematic cytokine analyses in larger samples to clarify the molecular pathways linking apelin dynamics with muscle recovery. Second, the 4–6-week observation window is unlikely to capture the full trajectory of muscular hypertrophy or the longer-term settling curve of circulating apelin. Longer follow-up (≥ 3–6 months) is needed to determine whether the early molecular signal we describe translates into sustained morphological and functional gains. Third, we did not enrol an age-matched healthy control group; consequently, baseline sarcopenia severity and ‘normal’ apelin reference ranges could not be defined within the same experimental framework, and recovery benchmarks remain provisional. Finally, although all participants received standard hospital meals and, when indicated, high-protein supplementation, the present study was not designed to dissect the relative contributions of nutritional optimisation *versus* exercise intensity to apelin dynamics. Future multicentre trials should therefore employ larger, stratified cohorts that include non-stroke controls, randomise nutritional support and graduated exercise loads, and extend follow-up beyond discharge, so that apelin’s utility as a precision biomarker—and potential therapeutic target—can be rigorously validated across diverse rehabilitation settings. Additionally, some patients in this study did not show a specific difference in their apelin levels despite significant improvement in physical function, which should be addressed in future studies. This could be addressed, for example, by introducing another indicative metric to measure the effectiveness of an exercise program. Finally, this study had a relatively short follow-up period. The length of stay in rehabilitation facilities is an important factor associated with an increase in skeletal muscle mass, and previous studies have shown that physical function and strength improve after several weeks to months of resistance training. In one study, there was no increase in SMI after 2 months of rehabilitation, but SMI increased after 3 months in older patients with stroke (Vinel et al., 2018). Therefore, the 4–6 week duration of our study may not have been sufficient to adequately capture changes in apelin levels and SMI and to explore the correlations between apelin and other outcomes. Therefore, future studies with longer follow-up periods and continuous rehabilitation are warranted. In addition, although we enrolled both ischemic (*n* = 20) and hemorrhagic (*n* = 3) stroke patients, the highly unbalanced distribution between these two etiologies precluded meaningful statistical analysis comparing their respective recovery trajectories. This is an important limitation, as stroke etiology may influence neuroplasticity, rehabilitation responsiveness, and systemic inflammatory profiles. Future studies involving larger and more balanced cohorts should investigate whether apelin expression and functional outcomes vary meaningfully by stroke subtype.

In conclusion, patients with stroke are at increased risk of skeletal muscle loss due to paralysis, which may progress to “post-stroke-associated sarcopenia” as a consequence of reduced physical activity and functional decline. Our study demonstrated significant improvements in functional recovery and psychosocial outcomes, as evidenced by increases in SMI following rehabilitation therapy. Importantly, the increase in blood apelin concentrations after rehabilitation correlated with these functional enhancements, whereas baseline albumin levels were associated with a rehabilitation-induced increase in apelin levels. These findings indicate that apelin may serve as a valuable biomolecular marker for post-stroke-assiciated sarcopenia and as an indicator of rehabilitation outcomes.

Beyond its role as a circulating biomarker, apelin has also emerged as a promising therapeutic candidate for age-related muscle decline and vascular dysfunction. Experimental studies have shown that exogenous apelin administration promotes skeletal muscle regeneration, enhances mitochondrial biogenesis, and counteracts age-associated sarcopenia and insulin resistance (Besse-Patin et al., 2014; Vinel et al., 2018). Mechanistically, apelin enhances satellite cell proliferation and activates AMPK/PGC-1*α* signalling, improving both metabolic and contractile muscle function (Luo et al., 2021). Additionally, apelin exerts vasodilatory and anti-inflammatory effects *via* endothelial nitric oxide pathways, which may improve perfusion and neuromuscular recovery in stroke patients (Mughal & O’Rourke, 2018). Furthermore, exploratory cytokine profiling from a single participant illustrated possible immunological adaptations accompanying apelin elevation. These findings should be interpreted as hypothesis-generating and warrant confirmation in larger, systematically profiled cohorts.

Taken together, our findings highlight apelin as a potential biomolecular marker linking nutritional status, muscle remodeling, and functional recovery in post-stroke–associated sarcopenia. Further studies with larger samples and longitudinal cytokine profiling are needed to validate these relationships and clarify apelin’s long-term role in rehabilitation outcomes.

## Supplemental Information

10.7717/peerj.20820/supp-1 Supplemental Information 1 Clinical raw data All the clinical measurements from Figs. 2 and 4 including blood apelin level before and after rehabilitation of stroke patients.

10.7717/peerj.20820/supp-2 Supplemental Information 2 Cytokine array raw data Raw measurements of cytokine array described in Fig. 3.

10.7717/peerj.20820/supp-3 Supplemental Information 3 STROBE checklist

## Additional Information and Declarations

### Data Availability

The following information was supplied regarding data availability: The raw measurements are available in the Supplemental Files.

## References

[ref-1] Beaudart C, Zaaria M, Pasleau F, Reginster JY, Bruyere O (2017). Health outcomes of sarcopenia: a systematic review and meta-analysis. PLOS ONE.

[ref-2] Besse-Patin A, Montastier E, Vinel C, Castan-Laurell I, Louche K, Dray C, Daviaud D, Mir L, Marques MA, Thalamas C, Valet P, Langin D, Moro C, Viguerie N (2014). Effect of endurance training on skeletal muscle myokine expression in obese men: identification of apelin as a novel myokine. International Journal of Obesity.

[ref-3] Bouassida A, Zalleg D, Bouassida S, Zaouali M, Feki Y, Zbidi A, Tabka Z (2006). Leptin, its implication in physical exercise and training: a short review. Journal of Sports Science and Medicine.

[ref-4] Carod-Artal FJ, Egido JA (2009). Quality of life after stroke: the importance of a good recovery. Cerebrovascular Diseases.

[ref-5] Castan-Laurell I, Dray C, Attane C, Duparc T, Knauf C, Valet P (2011). Apelin, diabetes, and obesity. Endocrine.

[ref-6] Chen LK, Woo J, Assantachai P, Auyeung TW, Chou MY, Iijima K, Jang HC, Kang L, Kim M, Kim S, Kojima T, Kuzuya M, Lee JSW, Lee SY, Lee WJ, Lee Y, Liang CK, Lim JY, Lim WS, Peng LN, Sugimoto K, Tanaka T, Won CW, Yamada M, Zhang T, Akishita M, Arai H (2020). Asian Working Group for Sarcopenia: 2019 consensus update on sarcopenia diagnosis and treatment. Journal of the American Medical Directors Association.

[ref-7] Collin C, Wade D (1990). Assessing motor impairment after stroke: a pilot reliability study. Journal of Neurology, Neurosurgery and Psychiatry.

[ref-8] Cruz-Jentoft AJ, Bahat G, Bauer J, Boirie Y, Bruyere O, Cederholm T, Cooper C, Landi F, Rolland Y, Sayer AA, Schneider SM, Sieber CC, Topinkova E, Vandewoude M, Visser M, Zamboni M, Writing Group for the European Working Group on Sarcopenia in Older P, The Extended Group for E (2019). Sarcopenia: revised European consensus on definition and diagnosis. Age Ageing.

[ref-9] Demeurisse G, Demol O, Robaye E (1980). Motor evaluation in vascular hemiplegia. European Neurology.

[ref-10] Dent E, Morley JE, Cruz-Jentoft AJ, Arai H, Kritchevsky SB, Guralnik J, Bauer JM, Pahor M, Clark BC, Cesari M, Ruiz J, Sieber CC, Aubertin-Leheudre M, Waters DL, Visvanathan R, Landi F, Villareal DT, Fielding R, Won CW, Theou O, Martin FC, Dong B, Woo J, Flicker L, Ferrucci L, Merchant RA, Cao L, Cederholm T, Ribeiro SML, Rodríguez-Mañas L, Anker SD, Lundy J, Gutiérrez Robledo LM, Bautmans I, Aprahamian I, Schols J, Izquierdo M, Vellas B (2018). International Clinical Practice Guidelines for Sarcopenia (ICFSR): screening, diagnosis and management. The Journal of Nutrition, Health and Aging.

[ref-11] Docherty S, Harley R, McAuley JJ, Crowe LAN, Pedret C, Kirwan PD, Siebert S, Millar NL (2022). The effect of exercise on cytokines: implications for musculoskeletal health: a narrative review. BMC Sports Science, Medicine and Rehabilitation.

[ref-12] Dray C, Debard C, Jager J, Disse E, Daviaud D, Martin P, Attane C, Wanecq E, Guigne C, Bost F, Tanti JF, Laville M, Vidal H, Valet P, Castan-Laurell I (2010). Apelin and APJ regulation in adipose tissue and skeletal muscle of type 2 diabetic mice and humans. American Journal of Physiology-Endocrinology and Metabolism.

[ref-13] Espárrago Llorca G, Castilla-Guerra L, Fernández Moreno MC, Ruiz Doblado S, Jiménez Hernández MD (2015). Post-stroke depression: an update. Neurologia.

[ref-14] Fedewa MV, Hathaway ED, Ward-Ritacco CL, Williams TD, Dobbs WC (2018). The effect of chronic exercise training on leptin: a systematic review and meta-analysis of randomized controlled trials. Sports Medicine.

[ref-15] Hashida R, Nakano D, Matsuse H, Yoshio S, Tsutsumi T, Kawaguchi M, Koya S, Hirota K, Tajima H, Sumida Y, Kanto T, Kawaguchi T, Hiraoka K (2023). A low-intensity 10-min resistance exercise program that ameliorated hepatic fibrosis indices and altered G-CSF/IP-10/PDGF-BB in a patient with nonalcoholic fatty liver disease: a case report. JGH Open.

[ref-16] Huo T, Guo Y, Shenkman E, Muller K (2018). Assessing the reliability of the short form 12 (SF-12) health survey in adults with mental health conditions: a report from the wellness incentive and navigation (WIN) study. Health and Quality of Life Outcomes.

[ref-17] Iolascon G, Di Pietro G, Gimigliano F, Mauro GL, Moretti A, Giamattei MT, Ortolani S, Tarantino U, Brandi ML (2014). Physical exercise and sarcopenia in older people: position paper of the Italian Society of Orthopaedics and Medicine (OrtoMed). Clinical Cases in Mineral & Bone Metabolism.

[ref-18] Janssen I, Shepard DS, Katzmarzyk PT, Roubenoff R (2004). The healthcare costs of sarcopenia in the United States. Journal of the American Geriatrics Society.

[ref-19] Kaya O (2016). Effect of a four-week exercise program on the secretion of IFN-*γ*, TNF-*α*, IL-2 and IL-6 cytokines in elite Taekwondo athletes. Biomedical Reports.

[ref-20] Kim G, Lee SE, Jun JE, Lee YB, Ahn J, Bae JC, Jin SM, Hur KY, Jee JH, Lee MK, Kim JH (2018). Increase in relative skeletal muscle mass over time and its inverse association with metabolic syndrome development: a 7-year retrospective cohort study. Cardiovascular Diabetology.

[ref-21] Kim M, Shinkai S, Murayama H, Mori S (2015). Comparison of segmental multifrequency bioelectrical impedance analysis with dual-energy X-ray absorptiometry for the assessment of body composition in a community-dwelling older population. Geriatrics & Gerontology International.

[ref-22] Kraemer RR, Chu H, Castracane VD (2002). Leptin and exercise. Experimental Biology and Medicine.

[ref-23] Krupp LB, LaRocca NG, Muir-Nash J, Steinberg AD (1989). The fatigue Severity Scale. Application to patients with multiple sclerosis and systemic lupus erythematosus. Archives of Neurology.

[ref-24] Kryger AI, Andersen JL (2007). Resistance training in the oldest old: consequences for muscle strength, fiber types, fiber size, and MHC isoforms. Scandinavian Journal of Medicine & Science in Sports.

[ref-25] Kwak SE, Cho SC, Bae JH, Lee J, Shin HE, Di Zhang D, Lee YI, Song W (2019). Effects of exercise-induced apelin on muscle function and cognitive function in aged mice. Experimental Gerontology.

[ref-26] Kwon JH, Moon KM, Min KW (2020). Exercise-induced myokines can explain the importance of physical activity in the elderly: an overview. Healthcare.

[ref-27] Lu L, Mao L, Feng Y, Ainsworth BE, Liu Y, Chen N (2021). Effects of different exercise training modes on muscle strength and physical performance in older people with sarcopenia: a systematic review and meta-analysis. BMC Geriatrics.

[ref-28] Luo J, Liu W, Feng F, Chen L (2021). Apelin/APJ system: a novel therapeutic target for locomotor system diseases. European Journal of Pharmacology.

[ref-29] Lupancu TJ, Eivazitork M, Hamilton JA, Achuthan AA, Lee KM-C (2023). CCL17/TARC in autoimmunity and inflammation—not just a T-cell chemokine. Immunology & Cell Biology.

[ref-30] MacIntosh BJ, Edwards JD, Kang M, Cogo-Moreira H, Chen JL, Mochizuki G, Herrmann N, Swardfager W (2017). Post-stroke fatigue and depressive symptoms are differentially related to mobility and cognitive performance. Frontiers in Aging Neuroscience.

[ref-31] Małkowska P, Sawczuk M (2023). Cytokines as biomarkers for evaluating physical exercise in trained and non-trained individuals: a narrative review. International Journal of Molecular Sciences.

[ref-32] Manrique-Espinoza B, Salinas-Rodriguez A, Rosas-Carrasco O, Gutierrez-Robledo LM, Avila-Funes JA (2017). Sarcopenia is associated with physical and mental components of health-related quality of life in older adults. Journal of the American Medical Directors Association.

[ref-33] Miranda-Cantellops N, Tiu TK (2024). Berg balance testing. StatPearls.

[ref-34] Mughal A, O’Rourke ST (2018). Vascular effects of apelin: mechanisms and therapeutic potential. Pharmacology & Therapeutics.

[ref-35] Naess H, Waje-Andreassen U, Thomassen L, Nyland H, Myhr KM (2006). Health-related quality of life among young adults with ischemic stroke on long-term follow-up. Stroke.

[ref-36] Norman K, Stobaus N, Pirlich M, Bosy-Westphal A (2012). Bioelectrical phase angle and impedance vector analysis–clinical relevance and applicability of impedance parameters. Clinical Nutrition.

[ref-37] Ogawa T, Suenaga M (2021). Elderly patients after stroke increase skeletal muscle mass by exercise therapy in rehabilitation wards. Journal of Stroke and Cerebrovascular Diseases.

[ref-38] Paciaroni M, Acciarresi M (2019). Poststroke fatigue. Stroke.

[ref-39] Pournajaf S, Pellicciari L, Proietti S, Agostini F, Gabbani D, Goffredo M, Damiani C, Franceschini M (2023). Which items of the modified Barthel Index can predict functional independence at discharge from inpatient rehabilitation? A secondary analysis retrospective cohort study. International Journal of Rehabilitation Research.

[ref-40] Rhind SG, Shek PN, Shinkai S, Shephard RJ (1996). Effects of moderate endurance exercise and training on in vitro lymphocyte proliferation, interleukin-2 (IL-2) production, and IL-2 receptor expression. European Journal of Applied Physiology and Occupational Physiology.

[ref-41] Roberts HC, Denison HJ, Martin HJ, Patel HP, Syddall H, Cooper C, Sayer AA (2011). A review of the measurement of grip strength in clinical and epidemiological studies: towards a standardised approach. Age Ageing.

[ref-42] Ryan AS, Ivey FM, Prior S, Li G, Hafer-Macko C (2011). Skeletal muscle hypertrophy and muscle myostatin reduction after resistive training in stroke survivors. Stroke.

[ref-43] Scherbakov N, von Haehling S, Anker SD, Dirnagl U, Doehner W (2013). Stroke induced Sarcopenia: muscle wasting and disability after stroke. International Journal of Cardiology.

[ref-44] Scherbakov N, Sandek A, Doehner W (2015). Stroke-related sarcopenia: specific characteristics. Journal of the American Medical Directors Association.

[ref-45] Shen Y, Shi Q, Nong K, Li S, Yue J, Huang J, Dong B, Beauchamp M, Hao Q (2023). Exercise for sarcopenia in older people: a systematic review and network meta-analysis. Journal of Cachexia, Sarcopenia and Muscle.

[ref-46] Shiraishi A, Yoshimura Y, Wakabayashi H, Tsuji Y (2018). Prevalence of stroke-related sarcopenia and its association with poor oral status in post-acute stroke patients: implications for oral sarcopenia. Clinical Nutrition.

[ref-47] Su Y, Yuki M, Otsuki M (2020). Prevalence of stroke-related sarcopenia: a systematic review and meta-analysis. Journal of Stroke and Cerebrovascular Diseases.

[ref-48] Tanaka S, Ando K, Kobayashi K, Seki T, Hamada T, Machino M, Ota K, Morozumi M, Kanbara S, Ito S, Ishiguro N, Hasegawa Y, Imagama S (2019). Low bioelectrical impedance phase angle is a significant risk factor for frailty. BioMed Research International.

[ref-49] Tatemoto K, Hosoya M, Habata Y, Fujii R, Kakegawa T, Zou MX, Kawamata Y, Fukusumi S, Hinuma S, Kitada C, Kurokawa T, Onda H, Fujino M (1998). Isolation and characterization of a novel endogenous peptide ligand for the human APJ receptor. Biochemical and Biophysical Research Communications.

[ref-50] The GBD 2016 Lifetime Risk of Stroke Collaborators (2018). Global, regional, and country-specific lifetime risks of stroke, 1990 and 2016. The New England Journal of Medicine.

[ref-51] Tolea MI, Chrisphonte S, Galvin JE (2018). Sarcopenic obesity and cognitive performance. Clinical Interventions in Aging.

[ref-52] Trøseid M, Lappegård KT, Claudi T, Damås JK, Mørkrid L, Brendberg R, Mollnes TE (2004). Exercise reduces plasma levels of the chemokines MCP-1 and IL-8 in subjects with the metabolic syndrome. European Heart Journal.

[ref-53] Vinel C, Lukjanenko L, Batut A, Deleruyelle S, Pradere JP, Le Gonidec S, Dortignac A, Geoffre N, Pereira O, Karaz S, Lee U, Camus M, Chaoui K, Mouisel E, Bigot A, Mouly V, Vigneau M, Pagano AF, Chopard A, Pillard F, Guyonnet S, Cesari M, Burlet-Schiltz O, Pahor M, Feige JN, Vellas B, Valet P, Dray C (2018). The exerkine apelin reverses age-associated sarcopenia. Nature Medicine.

[ref-54] Wang X, Yi X, Tang D (2021). Regular aerobic exercise activates PDGF-BB/PDGFR-*β* signaling and modulates the inflammatory-anti-inflammatory balance in diet-induced obese mice. Obesity Research & Clinical Practice.

[ref-55] Ware Jr J, Kosinski M, Keller SD (1996). A 12-item short-form health survey: construction of scales and preliminary tests of reliability and validity. Medical Care.

[ref-56] Winstein CJ, Stein J, Arena R, Bates B, Cherney LR, Cramer SC, Deruyter F, Eng JJ, Fisher B, Harvey RL, Lang CE, MacKay-Lyons M, Ottenbacher KJ, Pugh S, Reeves MJ, Richards LG, Stiers W, Zorowitz RD, American Heart Association Stroke Council CoC, Stroke Nursing CoCC, Council on Quality of C, Outcomes R (2016). Guidelines for adult stroke rehabilitation and recovery: a guideline for healthcare professionals from the American Heart Association/American Stroke Association. Stroke.

[ref-57] Yoshimura Y, Bise T, Nagano F, Shimazu S, Shiraishi A, Yamaga M, Koga H (2018). Systemic inflammation in the recovery stage of stroke: its association with sarcopenia and poor functional rehabilitation outcomes. Progress in Rehabilitation Medicine.

[ref-58] Yoshimura Y, Wakabayashi H, Bise T, Nagano F, Shimazu S, Shiraishi A, Yamaga M, Koga H (2019). Sarcopenia is associated with worse recovery of physical function and dysphagia and a lower rate of home discharge in Japanese hospitalized adults undergoing convalescent rehabilitation. Nutrition.

[ref-59] Zhao H, Cheng R, Song G, Teng J, Shen S, Fu X, Yan Y, Liu C (2022). The effect of resistance training on the rehabilitation of elderly patients with sarcopenia: a meta-analysis. International Journal of Environmental Research and Public Health.

