# Supplementary material for: Role of apelin as a biomarker in functional recovery and post-stroke-associated sarcopenia: insights from rehabilitation therapy
Source: PeerJ. 2026 Mar 6;14:e20820. doi: 10.7717/peerj.20820 (PMC12970307; doi:10.7717/peerj.20820)
Supplement: Supplemental Information 3 [file peerj-14-20820-s003.pdf]

STROBE Statement—checklist of items that should be included in reports of observational studies

	Item No	Recommendation
**Title and abstract**	1	(*a*) Indicate the study's design with a commonly used term in the title or the abstract<br>Longitudinal cohort study as stated in the Abstract on page 3 and Materials & Methods on page 7 and 8.
		(*b*) Provide in the abstract an informative and balanced summary of what was done and what was found<br>Provided in Abstract on page 3.
**Introduction**		
Background/rationale	2	Explain the scientific background and rationale for the investigation being reported<br>Included in the Introduction on pages 5 and 6.
Objectives	3	State specific objectives, including any prespecified hypotheses<br>Included in the Introduction on pages 5 and 6.
**Methods**		
Study design	4	Present key elements of study design early in the paper<br>Included in the Materials & Methods on page 7 and 8.
Setting	5	Describe the setting, locations, and relevant dates, including periods of recruitment, exposure, follow-up, and data collection<br>Included in the Materials & Methods on page 7 and 8.
Participants	6	(*a*) *Cohort study*—Give the eligibility criteria, and the sources and methods of selection of participants. Describe methods of follow-up<br>*Case-control study*—Give the eligibility criteria, and the sources and methods of case ascertainment and control selection. Give the rationale for the choice of cases and controls<br>*Cross-sectional study*—Give the eligibility criteria, and the sources and methods of selection of participants<br>Included in the Materials & Methods on page 7 and 8.
		(*b*) *Cohort study*—For matched studies, give matching criteria and number of exposed and unexposed<br>*Case-control study*—For matched studies, give matching criteria and the number of controls per case<br>Not applicable.
Variables	7	Clearly define all outcomes, exposures, predictors, potential confounders, and effect modifiers. Give diagnostic criteria, if applicable<br>Included in the Materials & Methods on page 7 and 8.
Data sources/ measurement	8*	For each variable of interest, give sources of data and details of methods of assessment (measurement). Describe comparability of assessment methods if there is more than one group<br>Included in the Materials & Methods on page 10~13.
Bias	9	Describe any efforts to address potential sources of bias<br>Addressed in the Discussion on page 23.
Study size	10	Explain how the study size was arrived at<br>Included in the Figure 1.
Quantitative variables	11	Explain how quantitative variables were handled in the analyses. If applicable, describe which groupings were chosen and why

		Included in the Materials & Methods on page 10~13.
Statistical methods	12	(*a*) Describe all statistical methods, including those used to control for confounding<br>Included in the Materials & Methods on page 14.
		(*b*) Describe any methods used to examine subgroups and interactions<br>Included in the Materials & Methods on page 14.
		(*c*) Explain how missing data were addressed<br>Not applicable.
		(*d*) *Cohort study*—If applicable, explain how loss to follow-up was addressed<br>*Case-control study*—If applicable, explain how matching of cases and controls was addressed<br>*Cross-sectional study*—If applicable, describe analytical methods taking account of sampling strategy<br>Included in the Materials & Methods on page 14.
		(*e*) Describe any sensitivity analyses<br>Not applicable.

Results

Participants	13*	(a) Report numbers of individuals at each stage of study—eg numbers potentially eligible, examined for eligibility, confirmed eligible, included in the study, completing follow-up, and analysed<br>Included in the Figure 1.
		(b) Give reasons for non-participation at each stage<br>Included in the Figure 1.
		(c) Consider use of a flow diagram<br>Included in the Figure 1.
Descriptive data	14*	(a) Give characteristics of study participants (eg demographic, clinical, social) and information on exposures and potential confounders<br>Included in the Results on page 15.
		(b) Indicate number of participants with missing data for each variable of interest<br>Included in the Results on page 7.
		(c) *Cohort study*—Summarise follow-up time (eg, average and total amount)<br>Included in the Results on page 7.
Outcome data	15*	*Cohort study*—Report numbers of outcome events or summary measures over time
		Case-control study—Report numbers in each exposure category, or summary measures of exposure
		Cross-sectional study—Report numbers of outcome events or summary measures<br>Included in the Results on pages 7 and 8, and summarized in Tables 1 ~ 4.
Main results	16	(*a*) Give unadjusted estimates and, if applicable, confounder-adjusted estimates and their precision (eg, 95% confidence interval). Make clear which confounders were adjusted for and why they were included<br>Included in the Results on pages 18 and 19, and summarized in Tables 1 ~ 4
		(*b*) Report category boundaries when continuous variables were categorized<br>Included in the Results on pages 18 and 19, and summarized in Tables 1 ~ 4
		(*c*) If relevant, consider translating estimates of relative risk into absolute risk for a meaningful time period<br>Not applicable.
Other analyses	17	Report other analyses done—eg analyses of subgroups and interactions, and sensitivity

		analyses<br>Not applicable.
**Discussion**		
Key results	18	Summarise key results with reference to study objectives<br>Included in the Discussion on page 20.
Limitations	19	Discuss limitations of the study, taking into account sources of potential bias or imprecision. Discuss both direction and magnitude of any potential bias<br>Included in the Discussion on page 23 and 24.
Interpretation	20	Give a cautious overall interpretation of results considering objectives, limitations, multiplicity of analyses, results from similar studies, and other relevant evidence<br>Included in the Discussion on page 20 ~ 24.
Generalisability	21	Discuss the generalisability (external validity) of the study results<br>Included in the Discussion on page 24.
**Other information**		
Funding	22	Give the source of funding and the role of the funders for the present study and, if applicable, for the original study on which the present article is based<br>Provided in the text on pages 26.

*Give information separately for cases and controls in case-control studies and, if applicable, for exposed and unexposed groups in cohort and cross-sectional studies.

Note: An Explanation and Elaboration article discusses each checklist item and gives methodological background and published examples of transparent reporting. The STROBE checklist is best used in conjunction with this article (freely available on the Web sites of PLoS Medicine at http://www.plosmedicine.org/, Annals of Internal Medicine at http://www.annals.org/, and Epidemiology at http://www.epidem.com/). Information on the STROBE Initiative is available at www.strobe-statement.org.
